# Acute behavioral changes in a patient with frontal lobe lesion of uncertain etiology: acute event or chronic finding?

**DOI:** 10.1192/j.eurpsy.2026.11626

**Published:** 2026-07-31

**Authors:** M. M. Munoz, E. L. Bardón, C. P. C. Alonso, J. G. Ruiz

**Affiliations:** Psychiatry, Caule, León, Spain

## Abstract

**Introduction:**

A 60-year-old patient with no relevant medical history who had been working until three months ago (currently on medical leave). He is married and has two children. There is no history of psychiatric or psychological follow-up, and he has never received psychiatric treatment.

He denies any substance use. He presents to the Emergency Department referred for rapidly emerging behavioral disturbances (over the past 3 months) with no clear precipitating factor, characterized by disinhibition, excessive spending, and inappropriate language. The patient lacks insight into the changes he has experienced and their impact on his functioning and daily life.

**Objectives:**

To establish a possible causal relationship between the altered brain structure seen on neuroimaging and the patient’s clinical presentation.

To determine whether the neuroimaging findings are the result of a chronic or acute process.

**Methods:**

In the Emergency Department, blood and urine tests were normal. A CT scan (Images 1 and 2) revealed a right frontal lesion compatible with encephalomalacia or a porencephalic cyst, with no signs of acute change. No previous neuroimaging was available. Lumbar puncture and EEG results were normal.

Psychopathological evaluation showed the patient to be alert and oriented, with slightly expansive mood not reaching mania. Speech was coherent, with some paranoid ideation and suspiciousness. Disinhibition and excessive spending were observed, though without frank aggression. No anxiety, suicidal thoughts, or psychotic features were noted. Circadian rhythms were disrupted.

A provisional diagnosis of organic personality disorder was made. The patient was admitted to psychiatry and started on Olanzapine 10 mg

**Results:**

Clinical evolution has been poor despite joint psychiatric and neurological care. Neurology was consulted; no acute neurological condition was identified. The abrupt onset suggests a possible organic cause, but the lesion appears chronic on imaging. After two months of treatment, the patient remains clinically unchanged, with improved sleep as the only notable progress.

**Image 1:**
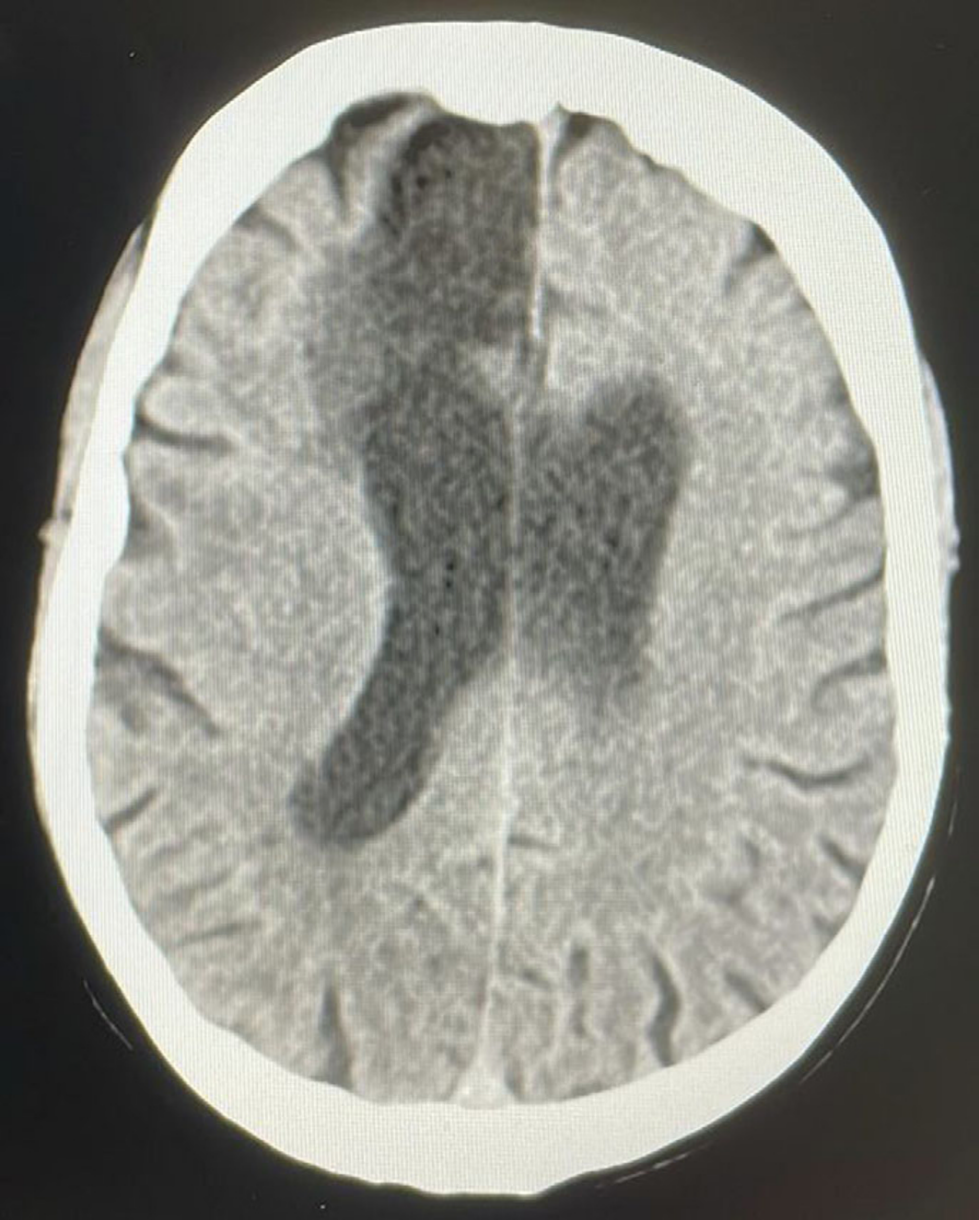

**Image 2:**
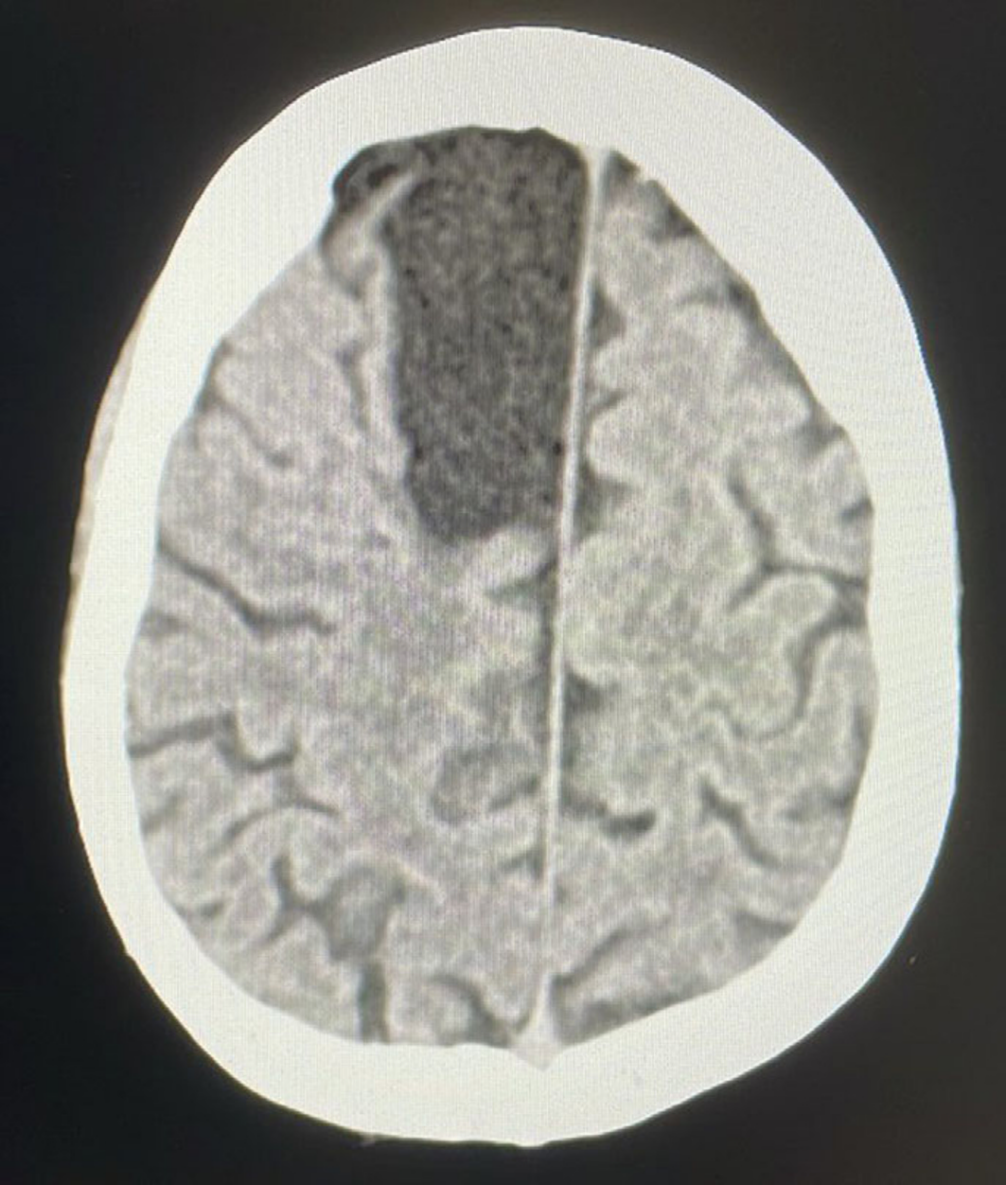

**Conclusions:**

Organic findings on neuroimaging often guide diagnosis. In this case, disinhibition aligns with a frontal lesion. However, the timing of onset suggests the symptoms may be unrelated to the imaging, which suggests a longstanding lesion. The patient was functioning normally until three months ago, and the cause of symptom onset remains unclear.

**Disclosure of Interest:**

None Declared

